# Treatment and Outcomes of Colorectal Cancer in Armenia: A Real-World Experience From a Developing Country

**DOI:** 10.1200/GO.20.00251

**Published:** 2020-08-12

**Authors:** Samvel Bardakhchyan, Sergo Mkhitaryan, Davit Zohrabyan, Liana Safaryan, Armen Avagyan, Lilit Harutyunyan, Jemma Arakelyan, Gevorg Tamamyan, Armen Tananyan

**Affiliations:** ^1^ Department of Oncology, Yerevan State Medical University, Yerevan, Armenia; ^2^ Adult Solid Tumors Chemotherapy Department, Haematology Center Yerevan State Medical University, Yerevan, Armenia; ^3^ Berd Military Hospital, Tavush, Armenia; ^4^ Department of Chemotherapy, Mikaelyan Institute of Surgery, Yerevan, Armenia; ^5^ Pediatric Cancer and Blood Disorders Center of Armenia, Hematology Center, Yerevan State Medical University, Yerevan, Armenia; ^6^ Department of Pediatric Oncology and Hematology, Yerevan State Medical University, Yerevan, Armenia

## Abstract

**PURPOSE:**

In Armenia, colorectal cancer (CRC) is one of the most frequently diagnosed cancers. It is in the third place by incidence. The aim of this study was to evaluate treatment and outcomes of CRC in Armenia during the last 9 years.

**MATERIALS AND METHODS:**

For this retrospective hospital-based study, we have collected data from two main oncology centers in Armenia: National Oncology Center and “Muratsan” Hospital of Yerevan State Medical University. The information about patients with CRC who were treated at these two centers between January 1, 2010 and July 1, 2018 was collected from the medical records. Log-rank test and Kaplan-Meier curves were used for survival analysis. Prognostic factors were identified by Cox regression.

**RESULTS:**

A total of 602 patients with CRC were involved in the final analysis. Median follow-up time was 37 months (range, 3-207 months). A total of 8.6% of patients had stage I, 32.9% stage II, 38.0% stage III, and 17.6% stage IV cancer; for 2.7% patients, the stage was unknown. The main independent prognostic factors for overall survival (OS) were tumor stage, grade, and histology. Adjuvant chemotherapy has been shown to improve survival in stage II colon cancer and stage III rectal but not in stage II rectal cancer. Radiotherapy did not yield survival improvement in stage II or III rectal cancer. Three- and 5-year OS rates were 62.9% and 51.8% for all stages combined and 79.7% and 68.5% for stages I-II, 62.5% and 48.4% for stage III, and 24.4% and 17% for stage IV respectively.

**CONCLUSION:**

As seen from our results, our survival rates are lower than those of the developed world. Additional research is needed to identify the underlying reasons and to improve patients’ treatment and outcomes in Armenia.

## INTRODUCTION

Colorectal cancer (CRC) is the third most commonly diagnosed cancer in males and the second in females worldwide, with an estimated 1.8 million cases and 881,000 deaths occurring in 2018.^1^ Approximately 21% of CRC cases are metastatic at diagnosis, and the 5-year overall survival (OS) is just 14% in this population.

**CONTEXT**

**Key Objective**
In this retrospective study, we tried to identify prognostic factors and analyze survival of patients with colorectal cancer (CRC) in Armenia.
**Knowledge Generated**
Armenian patients with CRC had shown lower survival rates compared with the developed world. In our study population, radiotherapy did not add survival for patients with stages II and III, whereas adjuvant chemotherapy was effective for patients with stage II and stage III colon cancer. Addition of targeted therapy with bevacizumab to standard chemotherapy did not bring any survival gain in patients with stage IV CRC.
**Relevance**
More thorough research is necessary to reveal the reasons behind our findings to improve treatment and outcomes of patients with CRC in Armenia.

CRC represents a heterogeneous group of dynamic biologic phenomena with differing sets of genetic events, accompanying immune responses, and influences of exogenous factors, providing a challenge for personalized therapeutic approaches. Prognostic factors continue to evolve and include tumor stage, grade, histology, location, microsatellite status, molecular markers, consensus molecular subtypes, and many others, with varying levels of significance.^2,3,4,5,6,7,8^ In the nonmetastatic setting, surgery remains the main treatment modality, whereas other treatment options (chemotherapy, radiotherapy) have shown additive benefit.^9,10,11,12^ In metastatic CRC (mCRC), the main treatment options are chemotherapy, targeted therapy, and, recently, immunotherapy.^9,12^ Targeted therapy selection is now based on presence or absence of several molecular markers (*RAS* and *BRAF* mutations)^7,13,14,15^ and also primary tumor sidedness, which recently was found to be a decisive factor.^9,13,16,17^ Responsiveness to immunotherapy is now predicted by microsatellite status (MSI).^7,9,18^ As our armamentarium of available therapies grows, in some studies the sequential use of various chemotherapeutic and targeted therapy agents has shown improvement in median survival of mCRC > 30 months.^19,20,21,22^

The situation is unfortunately different in developing countries because of the general unavailability of diagnostic tools, pathologic assessment, and molecular markers, as well as treatment modalities such as new targeted or immunotherapy options.^23-25^ In some of these countries, surgery is still the mainstay of treatment in most cases,^26^ and there are few articles studying effectiveness of chemotherapy and radiotherapy (RT). Nevertheless, these therapeutic modalities were found to improve survival when incorporated in the treatment plan.^25,27,28,29^ Five-year survival rates in some of these regions are much lower than in the developed world.^25^

Armenia is a small, developing country with a population of approximately 3 million people. The World Bank has ranked it as an upper middle income country.^30^ In Armenia, CRC is one of the most frequently diagnosed cancers: third among men (after lung and bladder cancer) and second among women (after breast cancer). In 2018, 682 new CRC cases were diagnosed. Thirty-three percent of patients were diagnosed as having metastatic disease.^31^

There are no national treatment guidelines for CRC in Armenia. However, in the nonmetastatic setting treatment generally consists of surgery, adjuvant chemotherapy (stages II and III), and adjuvant RT (for patients with stages II and III rectal cancer). In mCRC, chemotherapy with or without targeted therapy is the main treatment strategy. First-line chemotherapy regimens mostly contain fluorouracil (FU)/capecitabine with oxaliplatin, and second-line systemic therapy often consists of irinotecan-based regimens. Chemotherapy is not reimbursed by the government, and most treatment expenses, especially for medications, are covered by patients and their relatives. As a result, only a small number of patients with CRC can afford treatment with new, and potentially life-prolonging, systemic therapy (targeted therapy, immunotherapy).

To the best of our knowledge, there are no comprehensive studies on treatment and outcomes of CRC in Armenia. In this article, we tried to address these issues by analyzing the last 9 years of treatment of CRC in two oncology centers in our country.

## MATERIALS AND METHODS

### Patient Population

In this retrospective, hospital-based study, we collected information from the two main oncology centers of Armenia: the chemotherapy clinic of Muratsan hospital complex of Yerevan State Medical University and the National Center of Oncology. Patient characteristics and treatment histories were collected from the medical records. Patients who received treatment of CRC from January 01, 2010 until July 01, 2018 were included in this study.

Patients were divided into three subgroups by tumor location: right-sided CRC (RCC), left-sided CRC (LCC), and rectal cancer. Tumors located from the cecum to transverse colon were considered right-sided and from the splenic flexure to rectum were considered left-sided. CRC staging was done according to American Joint Committee on Cancer (AJCC), Union for International Cancer Control TNM 7th edition (2009),^32^ based on pathologic assessment and computed tomography (CT) scan.

Patient vital status was taken from medical records and from the local registry of the National Center of Oncology. This information was verified through contact with patients or their relatives by phone. Data cutoff was January 10, 2020. The patients who did not have follow-up information at the study end time were excluded.

Overall survival (OS), defined as time since diagnosis date until death/study end time, was calculated for all patients, and disease-free survival (DFS), defined as time since curative surgery date until recurrence/death/study end time, was calculated for patients with stage I-III disease who had undergone curative surgery; progression-free survival (PFS), defined as time since start of chemotherapy until progression/death/study end time, was calculated for patients with stage IV CRC who had not undergone curative surgery.

### Statistical Analysis

Log-rank tests and Kaplan-Meier curves were used for survival analysis. Univariate and multivariate Cox regression analysis was done, adjusting for baseline demographics and tumor characteristics. *P* value < .05 was deemed statistically significant in this study. All statistical analysis was done by SPSS version 20.0 (IBM Corporation, Chicago, IL).

## RESULTS

Eight hundred forty-four patients with CRC were identified. After excluding patients not matching our criteria, 602 patients remained. Of these, 312 (51.8%) were female. Median age at diagnosis was 58 years (range, 21-81 years). Median follow-up time was 37 months (range, 3-207 months). A total of 43.0% of patients had rectal cancer, 30.9% had LCC, and 26.1% had RCC. A total of 0.2% of patients had AJCC stage 0, 8.6% stage I, 32.9% stage II, 38.0% stage III, and 17.6% stage IV CRC, and 2.7% were unknown. A total of 89.4% of patients were diagnosed with pure adenocarcinoma, 7.6% mucinous, 2% signet ring cell, and 1% with adenosquamous carcinoma. DNA mismatch repair (MMR) protein status was checked by immunohistochemistry for only 1.7% of patients. *RAS* and *BRAF* status were also reported for a minority of cases (1.5% and 2.2%). Patients’ clinicopathological characteristics are shown in Table 1.

**TABLE 1:**
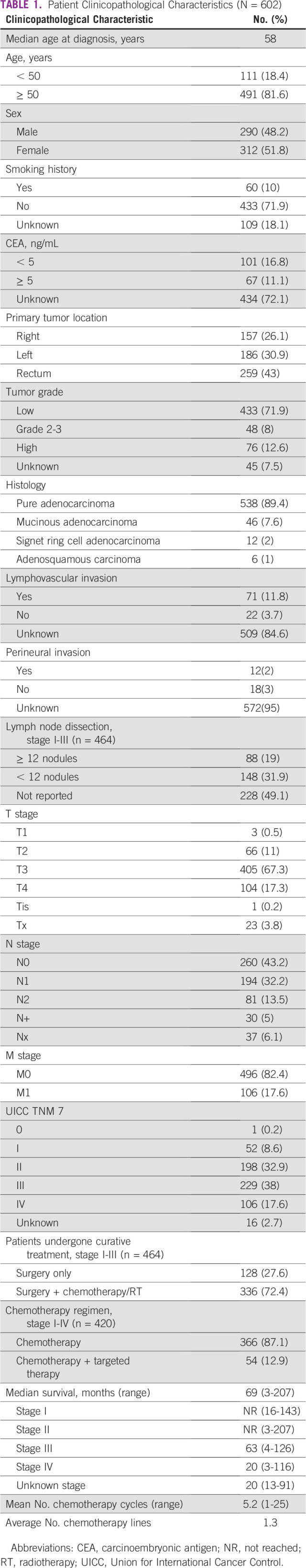
Patient Clinicopathological Characteristics (N = 602)

In our study population, patients with colon cancer with stage I-II disease underwent surgery with or without chemotherapy (55%), those with stage III disease underwent surgery with or without chemotherapy (87%), and patients with stage IV disease received chemotherapy with or without targeted therapy sometimes with curative surgery (27%).

Patients with stage I rectal cancer underwent surgery with or without chemotherapy (27%) and RT (27%). Those with stage II disease underwent surgery with or without chemotherapy (38.2%) and RT (54%). Those with stage III disease had surgery with or without chemotherapy (61%) and RT (44%). Patients with stage IV disease received chemotherapy with or without targeted therapy sometimes with curative surgery (12%).

The main independent prognostic factors for OS were tumor stage (*P* < .001), grade (*P* = .009), and histology (*P* = .011) in univariable and adjusted multivariable Cox regression analysis, whereas tumor location showed a trend toward significance (*P* = .063; patients with LCC living longer than patients with RCC and rectal cancers). Sex and age were not significant prognostic factors for OS in our study (*P* = .987 and *P* = .331, respectively; Table 2).

**TABLE 2:**
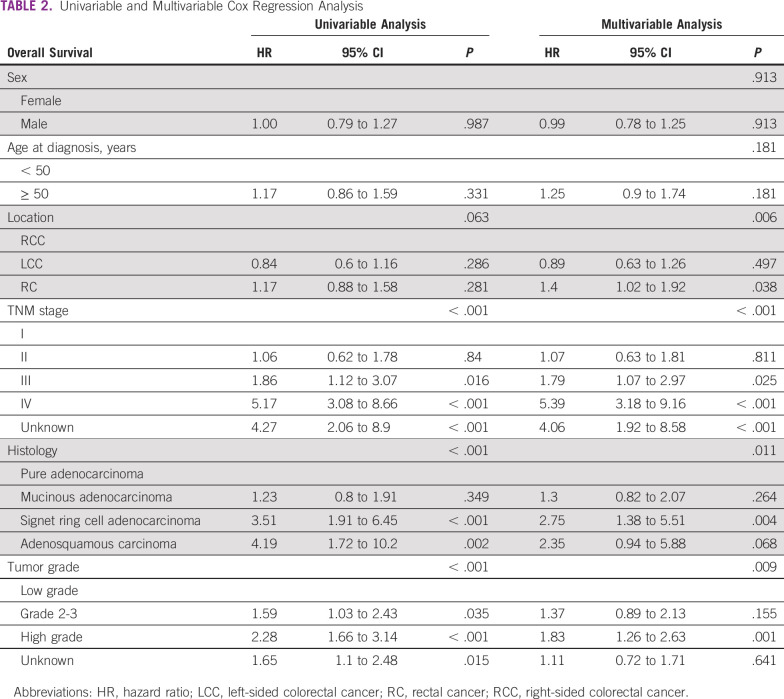
Univariable and Multivariable Cox Regression Analysis

**FIG 1:**
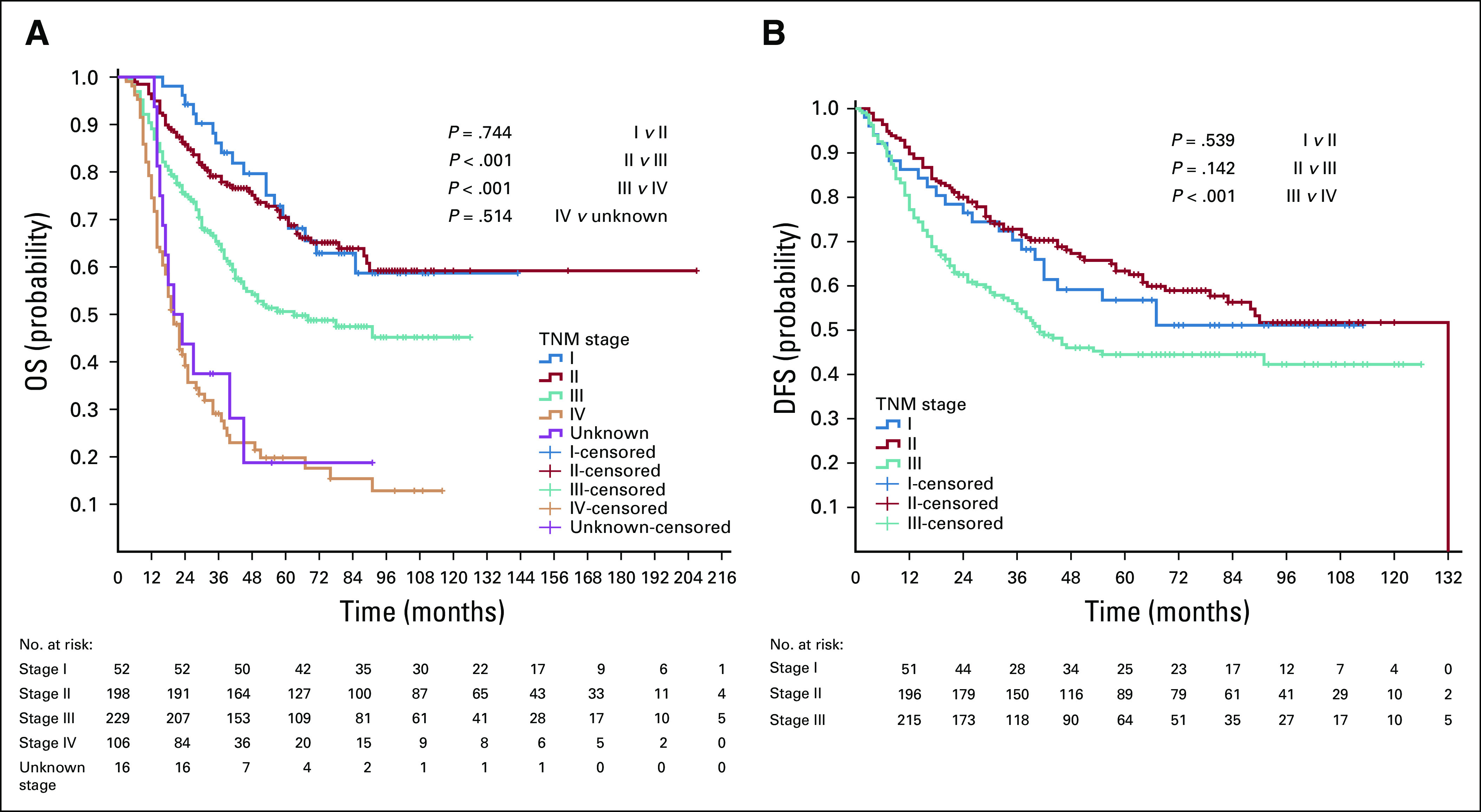
Kaplan-Meier survival curves for (A) overall survival (OS) for all stages, and (B) disease-free survival (DFS) for patients with stages I-III colorectal cancer.

Kaplan-Meier curves for OS for all stages and for DFS for stages I-III CRC are shown in Figure 1. The 3-year and 5-year OS rates were 79.7% and 68.5% for patients with stage I-II disease, 62.5% and 48.4% for patients with stage III disease, 24.4% and 17% for patients with stage IV disease, and 62.9% and 51.8% for all stages combined.

A total of 420 patients in our study group received chemotherapy. The mean number of chemotherapy cycles was 5.2 (range, 1-25). The most commonly used first-line chemotherapy regimens were modified FU + leucovorin + oxaliplatin (mFOLFOX6; 38.1%) or capecitabine + oxaliplatin (XELOX; 28.8%). Ninety-one patients (52.7%) received second-line chemotherapy, most with irinotecan-containing regimens.

### Nonmetastatic CRC

Four hundred eighty patients were diagnosed with nonmetastatic CRC. A total of 464 patients (96.7%) had undergone curative surgery. In this group, lymphovascular and perineural invasion were reported only for a few patients (16.2% and 5%, respectively). The number of lymph nodes examined was reported for 236 (50.9%) patients, of whom ≥ 12 lymph nodes were removed and examined in 19% and < 12 in 31.9%. Two hundred twenty-eight patients did not have a reported examined lymph node count (49.1%). When comparing OS among these 3 groups, we did not find any significant difference (*P* = .125).

### Nonmetastatic Colon Cancer

**FIG 2:**
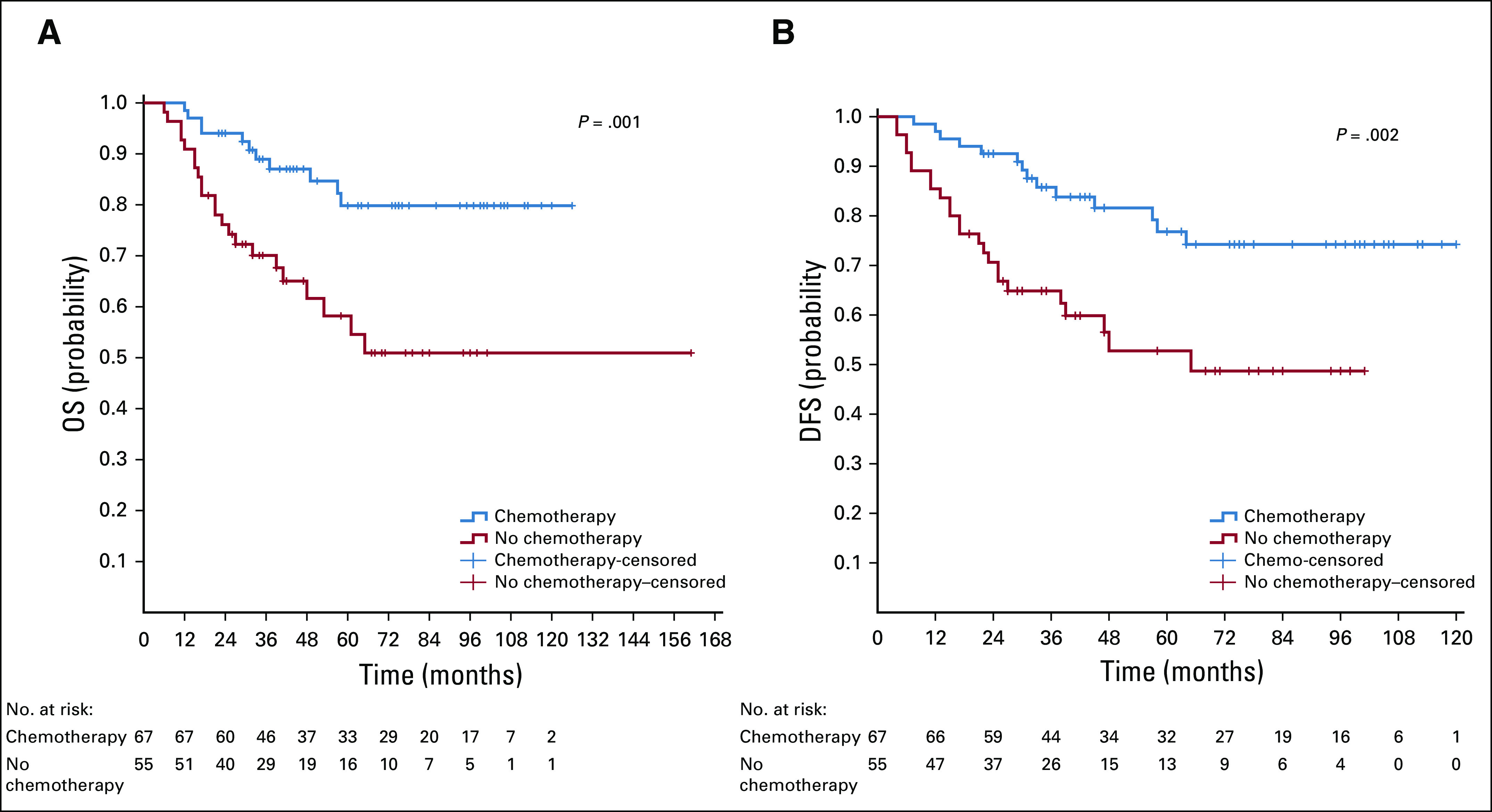
Kaplan-Meier survival curves for chemotherapy versus no chemotherapy in patients with stage II colon cancer. (A) Overall survival (OS) and (B) disease-free survival (DFS).

For the 122 patients with stage II colon cancer we found significant difference in OS and DFS in those who received adjuvant chemotherapy (54.9%) compared with those who did not (OS: not reached [NR] in both groups, *P* = .001; DFS: NR *v* 65 months, *P* = .002; Fig 2).

The number of patients with stage III colon cancer who did not receive adjuvant chemotherapy compared with those who received adjuvant chemotherapy was rather small (16 *v* 105); thus, statistical analysis was inappropriate.

### Nonmetastatic Rectal Cancer

**FIG 3:**
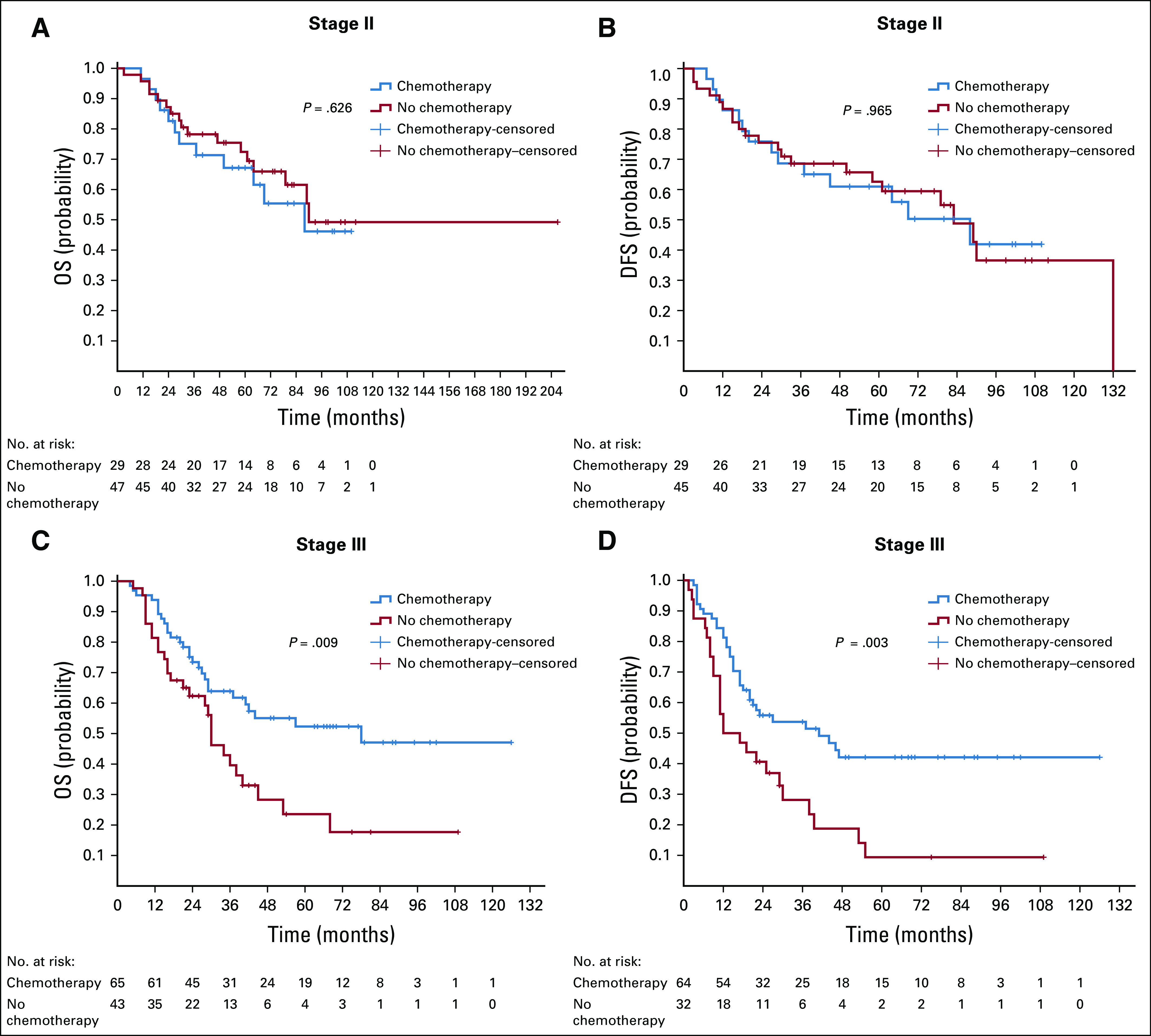
Kaplan-Meier survival curves for chemotherapy versus no chemotherapy in patients with stage II and III rectal cancer. (A) Stage II overall survival (OS) and (B) disease-free survival (DFS); (C) stage III OS and (D) DFS.

For the 76 patients with stage II rectal cancer we did not find any significant difference either in OS or DFS between those who received adjuvant chemotherapy (38.2%) and those who did not (OS: 88 *v* 90 months, *P* = .626; DFS: 88 *v* 83 months, *P* = .965). Among the 108 patients with stage III rectal cancer those who received adjuvant chemotherapy (60.2%) had better OS and DFS (OS: 78 *v* 30 months, *P* = .009; DFS: 41 *v* 12 months, *P* = .003; Fig 3).

**FIG 4:**
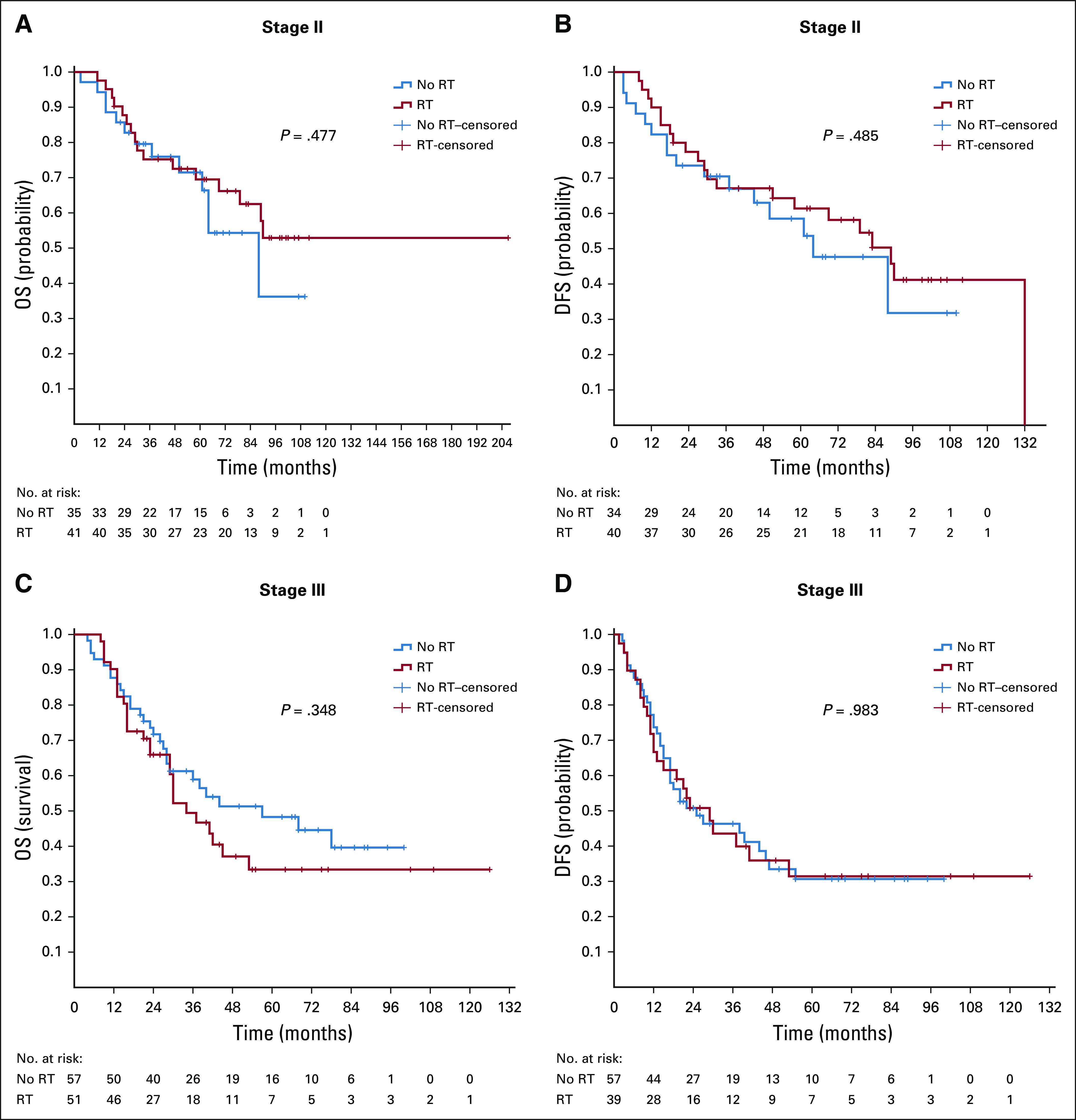
Kaplan-Meier survival curves for radiotherapy (RT) versus no RT in patients with stage II and III rectal cancer. (A) Stage II overall survival (OS) and (B) disease-free survival (DFS); (C) stage III OS and (D) DFS.

For stage II and III rectal cancer, 92 patients received adjuvant/neoadjuvant RT during their course of treatment, and 92 did not. The log-rank test did not show RT to be an independent prognostic factor for OS (stage II: *P* = .477; stage III: *P* = .348) or DFS (stage II: *P* = .485; stage III: *P* = .983) for both stages I and II. (Fig 4).

Of the 464 patients with stages I-III CRC who had undergone curative resection 44.8% developed disease recurrence. Recurrence was seen mainly within the first 4 years, with decreasing rates—first year, 36.5% of all recurrences, second year (26.4%), third year (13.5%), fourth year (12.5%), fifth year (3.4%), > 5 years (6.7%), and unknown (1%). The main site of recurrence was locoregional (21.6%), followed by multiple sites of recurrence (11.5%), liver (6.7%), and lungs (3.8%). For 45.7% of recurrences, the site was not reported.

### mCRC

In 106 patients with stage IV CRC, the sites of metastases included liver (44.3%), lungs (15.1%), and multiple (24.5%). The median OS was 20 months. The mean number of chemotherapy lines was 1.33, and the mean number of chemotherapy cycles received was six.

**FIG 5:**
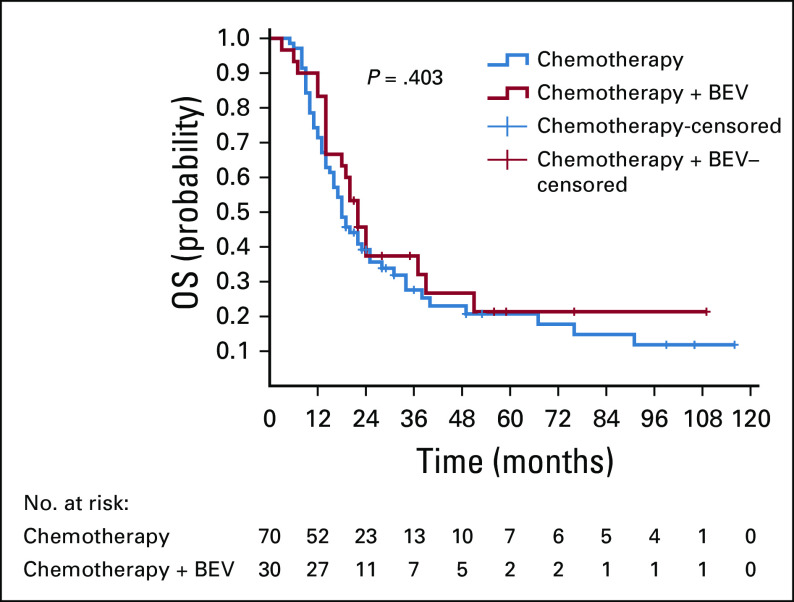
Kaplan-Meier overall survival (OS) curves for chemotherapy + bevacizumab (BEV) versus chemotherapy-only groups in patients with stage IV colorectal cancer.

Of 106 patients with stage IV CRC, 66% received only chemotherapy and 28.3% received chemotherapy combined with targeted therapy with bevacizumab. Cetuximab or other targeted agents were used only in a few cases (2.8%). Median OS in patients who received chemotherapy + bevacizumab was 22.0 months, compared with 18.0 months with chemotherapy alone (*P* = .403). Median PFS was 8 and 10.5 months, respectively (*P* = .503; Fig 5).

## DISCUSSION

The most influential prognostic factors for CRC survival in our study were TNM stage (*P* < .001) and tumor grade (*P* = .009; low grades surviving longer than high grades). These variables were found to be independent prognostic factors in many other studies.^23,25,33^

Tumor histology was also an important prognostic factor, with pure adenocarcinoma faring better than other adenocarcinoma subtypes (*P* = .011). Various studies are concordant with our findings showing patients with signet ring cell CRC to have worse survivability,^33^ whereas there is discrepancy regarding mucinous subtype. There are several reports that patients with mucinous histology had lower survival,^34^ while others failed to show any correlation with prognosis.^35^

Although some studies have identified sex as a prognostic factor for CRC survival,^36^ in our study we could not find any difference in OS by sex (*P* = .987). Age is considered to be a prognostic factor for CRC survival as well. There are several studies showing that younger patients are living longer than older patients,^37^ whereas others are showing opposite results.^38^ In our study, age at diagnosis failed to show significance regarding OS (*P* = .331).

According to our research, the 5-year OS rate for all stages was 53.3%—localized (68.5%), regional (48.4%), and mCRC (17%). These rates are consistent with those from other countries in our region, as well as other developing countries.^23,24,39^ For example, in Jordan, the 5-year survival rate was 58.2%—localized (72.1%), regional (53.8%), and metastatic stage (22.6%).^23^ However, the 5-year survival rate is higher compared with other of developing countries in our region.^25^ Although, our results are much lower than those of the developed world (eg, in the United States, the 5-year OS is 65%—localized (90%), regional (71%), and metastatic stages (14%).^40^

Our 5-year OS rates are much lower in early-stage (I-II) and regional-spread CRC, where surgery is the mainstay of treatment.^9^ One of probable explanation for this may be that many patients with stage I-II CRC who came to our clinics had received surgical treatment elsewhere and were admitted to those centers after disease recurrence. Thus, the patients with stage I-II CRC who received surgery at other surgical centers and who have not had disease recurrences are not represented, therefore decreasing our survival rates. The second reason is that in many cases, especially for patients with stage II CRC, we could not identify low- or high-risk groups.^9^ Recent studies have demonstrated the importance of histologic findings (ie, presence or absence of lymphovascular and perineural invasion^41,42^), which in our study population was reported in few cases. The number of surgically removed lymph nodes, which is also considered to be an important prognostic and decision-making factor,^4,43^ was omitted in 49.1% of patients with curative surgery. In 31.9% of patients, the number of lymph nodes removed was inadequate. However, when comparing OS for these three groups (omitted 49.1%; inadequate, 31.9%; and remaining [adequate], 19%), we found no significant difference. MSI/MMR status is now considered one of the most important molecular predictive factors and had a great role in adjuvant treatment decision making for patients with stage II colon cancer.^44,45,46^ Unfortunately, in our study population MSI/MMR status was checked only in a small subset of patients (1.7%), due to unavailability of the technique in Armenia until 2016 and high costs thereafter.

Our results demonstrated that patients with stage II colon cancer who received adjuvant chemotherapy did significantly better than those who received only surgery. However, the decision about giving adjuvant chemotherapy was mainly based on patients’ preference and not on histologic, molecular, or other risk factors. Therefore, we could not accurately distinguish between patients with low- and high-risk stage II disease and determine in which group adjuvant chemotherapy was beneficial. Literature review about this topic is discordant. Some studies showed no benefit with adjuvant chemotherapy for stage II colon cancer,^47^ and others recommended the use of adjuvant chemotherapy especially for patients with high-risk stage II disease, with small (∼5%) benefit.^9,48,49^

For rectal cancer, many guidelines are recommending the use of RT and chemotherapy in treatment of patients with stage II and III disease.^50^ In our study, half of the patients with stage II and III rectal cancer received radiotherapy during their initial treatment. We were not able to demonstrate an OS or DFS advantage for both stages. Our results are discordant with other studies showing improved OS and especially better DFS with neoadjuvant/adjuvant RT.^12,51,52^ In our study group, almost all RT was provided in the adjuvant setting. Therefore, we could not draw any conclusions about whether the situation would have been different if these patients had received neoadjuvant RT. Moreover, only a few patients received RT combined with chemotherapy. Furthermore, we did not have information about exact tumor location in the rectum (low or high) to see whether there was a subgroup of patients who benefited from RT.^52^ It is worth mentioning that staging was done mainly with CT scan. Pelvic magnetic resonance imaging or endorectal ultrasound were not used for patients with rectal cancer.

The role of adjuvant chemotherapy for stage II and III rectal cancer is not yet well established. Some trials have shown DFS and OS benefit from incorporation of adjuvant chemotherapy,^53^ whereas others have failed to show any improvement in survival.^54-58^ It is now generally recommended to use adjuvant chemotherapy after neoadjuvant chemotherapy and RT and surgery for these stages.^50^ In our study, chemotherapy failed to show any benefit in stage II, but it brought to significant survival gain in patients with stage III rectal cancer.

In stage IV CRC, our patients mainly received chemotherapy with or without targeted therapy and occasionally with curative or palliative surgery. The most commonly used chemotherapy regimens were FOLFOX/XELOX in the first line and irinotecan-based regimens in the second line. The most commonly incorporated targeted therapy was bevacizumab. In our study population, *RAS* and *BRAF* mutations and MSI/MMR status were checked, and thus anti-EGFR or immunotherapy drugs were used only in very few patients. Tumor sidedness, which is also considered to be an important factor for targeted therapy selection,^9,13,16,17^ was never taken into account. From 106 patients with mCRC, those who received chemotherapy plus targeted therapy with bevacizumab did not show survival benefit. Median OS in the chemotherapy + bevacizumab group was 22.0 months and in the chemotherapy-only group it was 18.0 months (*P* = .403). Median PFS was 8 and 10.5 months, respectively (*P* = .503). But numbers were rather small and not all patients received targeted therapy during the whole course of treatment.

Median OS of patients with stage IV CRC was 20 months. This is much lower when compared with that of the developed world, where the median OS for mCRC with the use of novel agents is now > 30 months.^17,19,20,22^ We believe that the main explanation for this is poor availability of drugs and especially new targeted agents. Only few patients could afford these novel agents and receive them during the whole course of treatment.

There are several limitations to our study. First it is a retrospective analysis, with a small number of patients from only two oncology centers in Armenia. Although these two oncology centers are the largest in the country and treat the most patients with cancer, results may differ in other centers and in the country as a whole. The study is also limited by the differences in treatment regimens received, given the absence of national guidelines for CRC treatment in Armenia.

In conclusion, survival of patients with CRC in Armenia is in line with other developing countries but is lower compared with the developed world. In our study we identified TNM stage, tumor grade, and histologic type as main prognostic factors for survival. RT did not show any improvement in OS/DFS for patients with stages II and III rectal cancer. Adjuvant chemotherapy has been shown to improve OS and DFS in stage II colon and stage III rectal cancer but not in stage II rectal cancer. Addition of targeted therapy with bevacizumab to standard chemotherapy did not bring OS advantage in patients with mCRC. Additional research is needed to identify the underlying reasons. We hope recent trends—better pathologic assessment, proper documentation and registration, availability of molecular markers, accessibility of new targeted drugs—may improve the outcomes.
